# Novel cuproptosis metabolism-related molecular clusters and diagnostic signature for Alzheimer’s disease

**DOI:** 10.3389/fmolb.2024.1478611

**Published:** 2024-10-24

**Authors:** Fang Jia, Wanhong Han, Shuangqi Gao, Jianwei Huang, Wujie Zhao, Zhenwei Lu, Wenpeng Zhao, Zhangyu Li, Zhanxiang Wang, Ying Guo

**Affiliations:** ^1^ Department of Neurosurgery, The Third Affiliated Hospital, Sun Yat-Sen University, Guangzhou, China; ^2^ Department of Neurosurgery, Xiamen Key Laboratory of Brain Center, The First Affiliated Hospital of Xiamen University, School of Medicine, Xiamen University, Xiamen, China

**Keywords:** Alzheimer’s disease, cuproptosis, molecular cluster, immune infiltration, gene signature

## Abstract

**Background:**

Alzheimer’s disease (AD) is a progressive neurodegenerative disorder with no effective treatments available. There is growing evidence that cuproptosis contributes to the pathogenesis of this disease. This study developed a novel molecular clustering based on cuproptosis-related genes and constructed a signature for AD patients.

**Methods:**

The differentially expressed cuproptosis-related genes (DECRGs) were identified using the DESeq2 R package. The GSEA, PPI network, GO, KEGG, and correlation analysis were conducted to explore the biological functions of DECRGs. Molecular clusters were performed using unsupervised cluster analysis. Differences in biological processes between clusters were evaluated by GSVA and immune infiltration analysis. The optimal model was constructed by WGCNA and machine learning techniques. Decision curve analysis, calibration curves, receiver operating characteristic (ROC) curves, and two additional datasets were employed to confirm the prediction results. Finally, immunofluorescence (IF) staining in AD mice models was used to verify the expression levels of risk genes.

**Results:**

GSEA and CIBERSORT showed higher levels of resting NK cells, M2 macrophages, naïve CD4^+^ T cells, neutrophils, monocytes, and plasma cells in AD samples compared to controls. We classified 310 AD patients into two molecular clusters with distinct expression profiles and different immunological characteristics. The C1 subtype showed higher abundance of cuproptosis-related genes, with higher proportions of regulatory T cells, CD8^+^T cells, and resting dendritic cells. We subsequently constructed a diagnostic model which was confirmed by nomogram, calibration, and decision curve analysis. The values of area under the curves (AUC) were 0.738 and 0.931 for the external datasets, respectively. The expression levels of risk genes were further validated in mouse brain samples.

**Conclusion:**

Our study provided potential targets for AD treatment, developed a promising gene signature, and offered novel insights for exploring the pathogenesis of AD.

## Introduction

Dementia is a syndrome characterized by cognitive decline, manifested by abnormalities in memory function, mental, behavioral, and personality. Alzheimer’s disease (AD), the most common dementia, impacts more than 42 million people worldwide and imposes a considerable burden on society (Ruthirakuhan et al., 2022). Early treatment and diagnosis are essential for AD. Nonetheless, the diagnosis of AD relies primarily on invasive and instrumental tests, and effective diagnostic biomarkers are lacking (Scheltens et al., 2016). Since the exact mechanism of AD is not precise, no effective strategies are available to retard the development of this disease (Khan et al., 2020).

Neuroinflammation is now considered an essential factor in the development of AD (Leng and Edison, 2021). Phenotypes targeting immune cells and crosstalk with specific cytokines in the brain may effectively attenuate the inflammatory response in AD (Liddelow et al., 2017). Previous studies have shown that many distinct cell death mechanisms mediate AD progression, for example, necroptosis, pyroptosis, ferroptosis, and apoptosis (Calvo-Rodriguez and Bacskai, 2021). In 2022, Tsvetkov et al. discovered a new copper-dependent cell death called cuproptosis (Tsvetkov et al., 2022). It is a non-apoptotic form of cell death dependent on cellular resorption and mitochondrial stress. It is marked by the absence of Fe-S cluster proteins and the accumulation of lipoylated proteins. Several studies have shown that mitochondrial malfunction plays a crucial role in AD (Ashleigh et al., 2023; Perez Ortiz and Swerdlow, 2019; Sharma et al., 2021). Therefore, we may infer that cuproptosis is closely related to AD progression. Nevertheless, the underlying mechanisms are not well understood. Meanwhile, increasing evidence suggests that cuproptosis plays a part in regulating the immune micro-environment in neurodegenerative diseases (Nie et al., 2023; Caetano-Silva et al., 2021; Ban et al., 2024). This study provided the first integrated analysis of cuproptosis-related genes (CRGs) between AD samples and normal controls. Potential targeted compounds for AD and transcription factors binding hub genes were identified. Unsupervised clustering and machine learning algorithms were employed to detect hub genes to predict the risk of Alzheimer’s disease. This gene signature was verified using calibration curves, nomograms, decision curve analysis, receiver operating characteristic (ROC) curves, and *in vitro* experiments. Furthermore, the associations between CRGs and infiltrating immune cells were investigated.

## Materials and methods

### Extraction of data

The mRNA representation profiles of AD and normal samples were downloaded from the Gene Expression Omnibus (GEO) database (Narayanan et al., 2014; McKay et al., 2019; Sood et al., 2015; Patel et al., 2019). The details of the datasets used are listed in Supplementary Table S1. The GSE33000 dataset contained expression profiles of 624 brain tissues, including 157 controls and 310 AD samples (Table 1). When multiple probes refer to a single gene, we take their average as the expression values. The “sva” R package was designed to eliminate batch effects and unwarranted variations. After removing the batch effects, these raw gene expression files were normalized and processed via the “affy” package. According to previous published literature, we obtained 19 cuproptosis-related genes (ATP7A, ATP7B, CDKN2A, DBT, DLD, DLAT, DLST, FDX1, GLS, GCSH, LIAS, LIPT1, LIPT2, MTF1, NLRP3, NFE2L2, PDHA1, PDHB and SLC31A1) (Tsvetkov et al., 2022).

**TABLE 1:** GSE33000 dataset clinical information.

Parameter	Subclass	Patients
Samples	Control	157
	AD	310
Age	>60	449
	≤60	175
Gender	Female	283
	Male	341

### Analysis of differentially expressed CRGs (DECRGs)

DECRGs between normal controls and AD samples in GSE33000 were screened out using the “limma” R package, with the criteria at |log_2_ fold change (FC)| > 1 and Bonferroni-p value <0.05. In addition, we also compared the gender differences of DECRGs in AD patients. The visualizations of results were accomplished using “pheatmap” and “ggplot2” packages. The Kyoto Encyclopedia of Genes and Genomes (KEGG) and Gene Ontology (GO) analyses of the DECRGs were performed using the “clusterProfiler” package and the Benjamini–Hochberg (BH) p-adjust Method. The following parameters were: q-value <0.05, *p*-value <0.05. The protein–protein interaction (PPI) network was structured with the linear STRING repository (https://string-db.org/), and 0.900 (the highest confidence) was set as the minimum required interaction score.

### Immune characteristics

CIBERSORT is a tool for accurately assessing the relative ratios of different cell subsets in tissues from the data provided. In recent years, different immune-ecological niches have been identified in the brain from which innate and adaptive immune cells can regulate brain function and perform repair (Castellani et al., 2023). The proportion of 22 immune-infiltrating cells in each sample was estimated from the GSE33000 dataset with the CIBERSORT algorithm. The total fraction of immune cells per sample is equal to 1. The empirically defined *p*-value of each piece for deconvolution was then determined. Samples with a *p*-value <0.05 were determined to be significant (Newman et al., 2015). A single-sample gene set enrichment analysis (ssGSEA) was conducted with the “GSVA” package to assess the composition of enriched immunity-related functions in each sample. The fractions of immune cells between normal controls and AD samples were visualized using a boxplot and heatmap.

### Correlation analysis

In addition, we explored the correlations and correlation coefficients between DECRGs using the “corrplot” and “circlize” packages. Spearman correlation analysis of DECRGs and immune infiltration was conducted with the “ggcorrplot” package. According to the correlation coefficients, interactions with a *p*-value less than 0.05 were assumed to be significant.

### Clustering of DECRGs, gene set variation analysis (GSVA)

Next, we conducted the unsupervised cluster analysis of 310 AD patients into clusters based on the gene expression profiles of the DECRGs by the “ConsensusClusterPlus” R package. Furthermore, we conducted principal component analysis (PCA) to assess the performance of clustering. GSVA was performed using the “GSVA” and “GSEABase” packages to investigate differences in the sets of enriched genes between different DECRG clusters (Haenzelmann et al., 2013). The referred sets were “c5. go.symbols”, “c2. cp.kegg.symbols”, and “c7. immunesigdb.v2023.1. Hs.symbols” obtained from the MSigDB database. Differences were considered significant when the absolute t-values were >2.

### Weighted gene co-expression network analysis (WGCNA)

Using the WGCNA software package, we performed two separate analyses for normal and AD patients based on the cuproptosis clustering and gene expression profiles in GSE33000 (Wan et al., 2018). First, we constructed an adjacency matrix using the topological overlap matrix (TOM) and soft threshold 7. The genes characterized by the model were then calculated, as well as the associations between the model and clinical phenotypes. Finally, the most relevant gene models were screened. Module significance (MS) indicated the relationship between modules and disease states. Gene significance (GS) was depicted as the relevance between genes and clinical traits. Module eigengene referred to the overall gene expression profile of each module.

### Construction of the diagnostic signature

Based on the results of WGCNA, we chose the intersected genes for further analysis with the “VennDiagram” package (version 1.7.2). The random forest (RF) algorithms, eXtreme Gradient Boosting (XGB), generalized linear models (GLM), and support vector machine (SVM) were employed to identify the most significant hub genes for AD risk. The RF algorithms were integrated with machine learning methods for determining the optimum amount of variables using multiple separate decision treaties (Rigatti, 2017). The SVM was used to locate the optimum variables by finding the minimum matching points of cross-validation errors. We performed the above machine learning techniques using the “caret”, “randomForest”, “kernlab”, “xgboost”, and “e1071″packages. We then evaluated the diagnostic performance of the above methods by root mean square errors (RMSE), boxplots of residuals, and ROC curves. Hence, the optimum model was identified and the top 5 significant genes were used as key diagnostic markers for AD risk. Finally, a nomogram was developed through multi-factor logistic regression for the five genes using the “rms” software package. Each gene was given a matching “Point” and the “Total Points” denoted the aggregate of the above gene points. The prediction capacity was evaluated using decision curve analysis (DCA), calibration, and ROC curves.

### Clinical correlation analysis

GSE118553, containing 100 controls and 301 AD brain tissues, was selected as the externally analyzed dataset. We explored the associations between these five predictive genes and clinical features, including gender (female n = 166, male n = 235) and age (>60 yrs n = 368, ≤60 yrs n = 33) (Table 2).

**TABLE 2:** The clinical information of GSE118553 dataset.

Parameter	Subclass	Patients
Samples	Control	100
	AD	301
Age	>60	368
	≤60	33
Gender	Female	166
	Male	235

### CeRNA networks and immune correlations

A combination of miRanda, miRDB, miRTarBase, and TargetScan databases were employed to predict the targeted microRNAs. The SpongeScan database was used to predict the matching lncRNAs. We finally constructed a ceRNA network based on the five genes using Cytoscape software (version 3.8.2). The “linkET” package was used to further explore the relationship between 22 immune cells and between these risk genes and the immune cells mentioned above.

### Prediction of interacting genes, transcription factors, and drugs

Gene interactions were predicted using the online GeneMANIA database. JASPAR is a publicly available multi-species repository of transcription factors (TF) (Rigden and Fernández, 2023). NetworkAnalyst is an extensive online portal for conducting analyses of gene expression data (Zhou et al., 2019). We identified topologically plausible TFs that tend to combine with these hub genes using the National Center for Biotechnology Information (NCBI) database and JASPAR on the NetworkAnalyst database. The relative profile score threshold was set at 80%. Moreover, we utilized the DGIdb database to predict compounds that might target these hub genes and presented the results with Cytoscape software (version 3.8.2).

### AD mouse model

The mice were purchased from GENEANDPEACE (Jiangsu, China). Male App/PS1 mice (AD groups) and wild-type mice (control groups) (10–12°weeks old) were caged (6 mice per cage) in air-conditioned chambers with a temperature of ∼26°C and received a light/dark cycle of 12 h for 7 days before the research. No drug tests were conducted. All mice were raised until 9 months of age in the animal facilities of Xiamen University. The animal experiment programs were performed in accordance with the Animal Protection Committee of Sun Yat-sen University and NIH Guide for the Care and Use of Laboratory Animals.

### Antibodies and immunofluorescence (IF)

The primary antibodies were CAMK4 (Proteintech, 13263-1-AP), GPI (Proteintech, 15171-1-AP), ITPKB (Proteintech, 12816-1-AP), CKMT1A (Proteintech, 15346-1-AP), and PCSK2 (Proteintech, 10553-1-AP). The fluorophore-labeled antibodies used were goat anti-mouse Alexa Fluor 549 (1:500; Abbkine) and goat anti-rabbit Alexa Fluor 488 (1:500; Abbkine). Brain tissues from wild-type and App mice were fixated with 4% paraformaldehyde, buried in paraffin, and sliced into four μm-sized sections using a microtome (MicromHm325, Thermo Scientific). The tissue slices were deparaffinized with dimethylbenzene, dehydrated with graded alcohol, and then heated in citrate buffer (pH 6.0). Following this, permeabilization with 0.4% Triton X-100 was performed for 30 min and closed with goat serum working solution (Wuhan, China) for two h after antigen repair. The slices were incubated overnight with the above primary antibodies at 4 °C and rinsed with PBS. Lastly, the sections were assayed in the dark with secondary antibodies for 1 hour at room temperature. The slices were mounted with 4′, 6 diamidino-2-phenylindole for nuclear staining. The images were captured using a confocal microscope (Nikon A1 + R, Tokyo, Japan) and analyzed with Image-Pro Plus 5.1 software. The percentage of positive cells and staining intensity were assessed semi-quantitatively by the pathologists.

### Ethics declarations

The authors confirm that all methods reported in this study were performed in accordance with relevant guidelines and regulations, including the ARRIVE guidelines (Percie du Sert et al., 2020).

### Statistics

We used the above packages in R software (version 4.3.1) for bioinformatics analyses and GraphPad Prism 9.5 software for *in vitro* experimental data analysis. Experimental results and data were expressed as mean ± standard deviation for a minimum of three independent experiments. The correlation was identified by Spearman correlation analysis. When making comparisons, the Wilcoxon rank-sum test applied to non-normally distributed variants, while the Student’s t-test worked for normally distributed variants. Two-tailed *p*-values less than 0.05 were considered significant.

## Results

### DECRGs in AD

**FIGURE 1:**
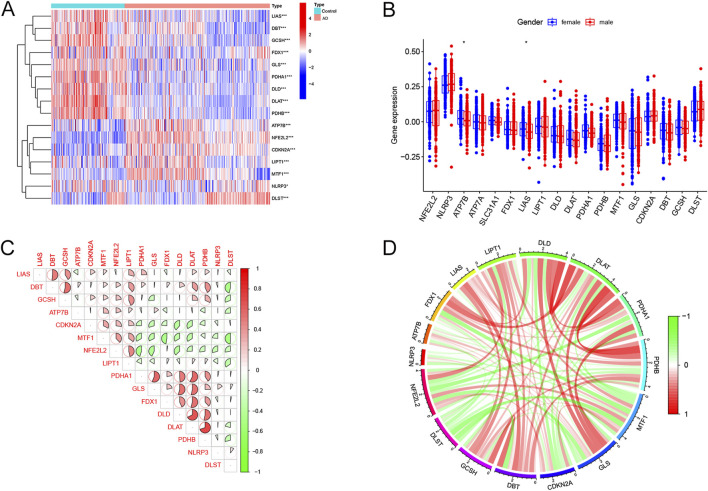
Expression characteristics of cuproptosis-related differentially-expressed genes in AD. **(A)** The heatmap exhibited the expression landscapes of 16 DECRGs between AD and normal controls. **(B)** The boxplot of DECRGs between female and male AD patients. **(C)** Correlation analysis of the 16DECRGs. The area of the pie diagram indicated the correlation coefficient. Red and green colors represented positive and negative correlations, respectively. **(D)** Gene relationship network circle diagram of 16 DECRGs. Green and red colors represented negative and positive correlations, respectively. The thickness of the line represents the closeness of the relationship (for all figures, *, *p* < 0.05, **, *p* < 0.01, ***, *p* < 0.001).

**FIGURE 2:**
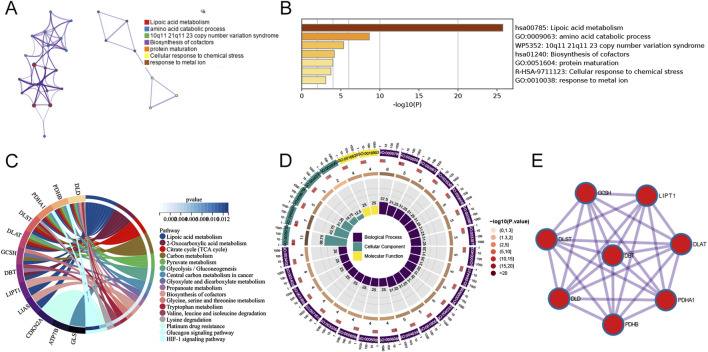
Functional enrichment analysis of 16 differentially-expressed cuproptosis-related genes. **(A)** A network of enriched terms. **(B)** Histogram of biological pathways based on *p* values. **(C)** The circle diagram of KEGG pathway analysis. **(D)** The circle diagram of GO enrichment analysis. **(E)** PPI network analysis with interaction scores setting >0.900.

There were 16 differentially expressed cuproptosis-related genes, including ten downregulated and six upregulated genes. The heatmap of DECRGs is displayed in Figure 1A. *NFE2L2*, *ATP7B*, *LIPT1*, *CDKN2A*, *MTF1*, and *DLST* were upregulated in AD, while the other genes were downregulated. Additionally, we analyzed the expression levels of CRGs between female and male AD patients. Only *ATP7B* and *LIAS* were identified as differentially expressed genes regarding gender (Figure 1B). The correlations between the 16 DECRGs are shown in Figures 1C, D. The expression levels of LIAS were associated with the levels of all the other 15 DECRGs. Functional enrichment analyses were then performed. We found that cellular energy metabolic processes, such as lipoic acid metabolism and amino acid catabolic process, were remarkably enriched (Figures 2A, B). Furthermore, some glycolysis-related processes were enriched, such as the acetyl-CoA metabolic process, TCA cycle, and 2-Oxocarboxylic acid metabolism (Figures 2C, D; Supplementary Tables S2, S3). These results revealed that DECRGs were strongly associated with the processes of mitochondrial aerobic respiration in AD. Finally, the PPI network identified eight hub genes from the DECRGs (Figure 2E).

### Immune infiltration and clustering analyses

**FIGURE 3:**
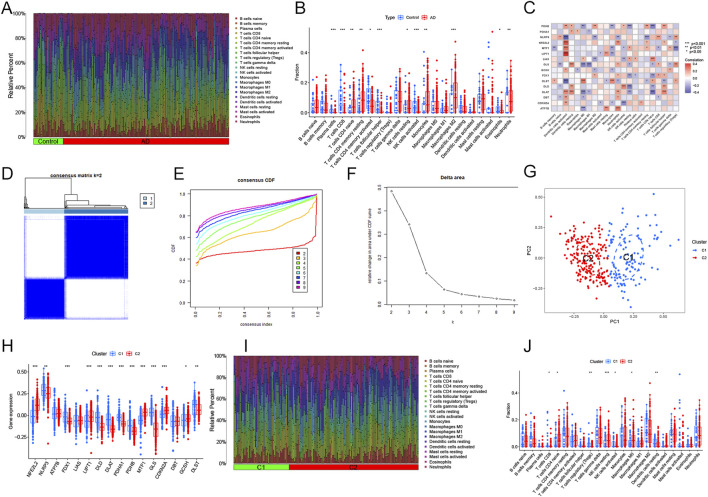
Immune characteristics and cuproptosis-related clusters in AD. **(A)** Relative abundance of the 22 immune cells in AD *versus* normal controls. **(B)** Box plot showing the difference in immune infiltration between AD and normal controls. *p < 0.05, **p < 0.01, ***p < 0.001. **(C)** Correlation analysis of the 16 DECRGs with immune infiltrating cells. **(D)** The consensus clustering matrix at k = 2. **(E)** Typical cumulative distribution function (CDF) curves at k = 2–9. **(F)** CDF delta area curves. **(G)** Principal component analysis (PCA) of the two clusters. Blue dots represent samples of Cluster 1, and red dots represent samples of Cluster 2. **(H)** The expression patterns of 16 key DECRGs between the two molecular clusters were shown in the heatmap. **(I)** Relative abundance of the 22 immune-infiltrated cells between the two molecular clusters. **(J)** Variations in immune infiltration between the two molecular clusters were shown in the boxplots. *p < 0.05, **p < 0.01 ***p < 0.001.

Figure 3A shows the immune cell infiltration in both AD and control samples. The results of ssGSEA showed that the abundance of resting NK cells, naïve CD4^+^ T cells, M2 macrophages, resting CD4^+^ memory T cells, neutrophils, and monocytes was remarkably increased in AD patients (Figure 3B). We speculated that changes in the immune microenvironment might be essential to AD development. Moreover, correlation analysis showed that all DECRGs were significantly associated with infiltrating immune cells (Figure 3C). Consensus clustering analysis showed the optimal stability of clusters with k = 2 (Figures 3D–F). Based on these results, we finally categorized the 310 AD patients into two subgroups: Cluster 1 and Cluster 2. PCA reduced the sample dimensions of the two clusters quite well (Figure 3G). To investigate the associations between cuproptosis and the two AD clusters, the expression profiles of 16 DECRGs between Clusters 1 and 2 were compared. The abundance of DECRGs was significantly different between the two subtypes (Figure 3H). Moreover, we explored the variations in immune infiltrating cells between the two subgroups. The bar plots showed the infiltrating levels of immune cells in the two clusters (Figure 3I). The results showed that activated NK cells, regulatory T cells, CD8^+^T cells, and resting dendritic cells were remarkably increased in Cluster 1. In contrast, M0 macrophages, M1 macrophages, and resting NK cells were significantly decreased compared to Cluster 2 (Figure 3J).

### Functional annotations

**FIGURE 4:**
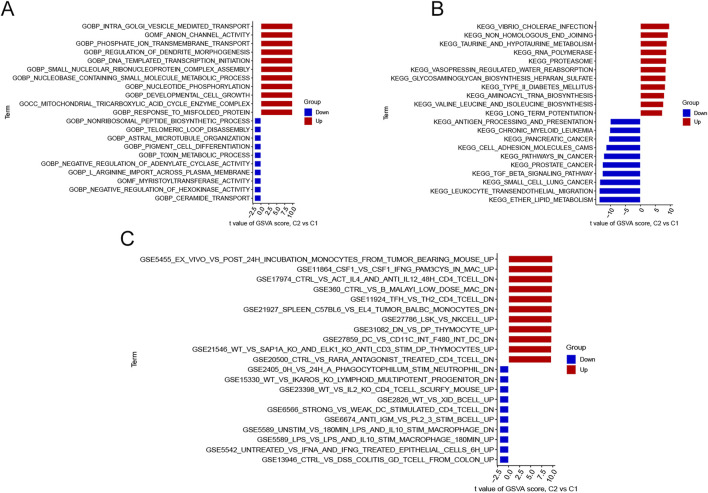
Biological differences between the two Clusters sorted by t-values and intersections of significant modules. **(A)** Differences in GO biological functions between the two Clusters. **(B)** Variations in KEGG pathways between the two Clusters. **(C)** Differences in cell states and perturbations within the immune system between the two Clusters.

GSVA analysis was employed to explore the differences in biological processes and functions between the two clusters (Figures 4A, B). The results indicated that response to misfolded protein, mitochondrial tricarboxylic acid cycle enzyme complexes and developmental cell growth, were upregulated in Cluster 2. These biological processes were consistent with the findings of previous studies in AD (Heneka et al., 2015; Oliver et al., 2020; Shoshan-Barmatz et al., 2018). Meanwhile, the non-ribosomal peptide biosynthetic process, toxin metabolic process, and ceramide transport were reinforced in Cluster 1, which were also closely linked to AD (Crivelli et al., 2021; Pugazhenthi et al., 2017; Zhao et al., 2020). KEGG terms showed that long-term potentiation, RNA polymerase and non-homologous end joining were enriched in Cluster 2, whereas ether lipid metabolism and TGF-β signaling pathway were significantly involved in Cluster 1. All of these pathways were markedly associated with AD progression (Reddy et al., 2018; Lourenco et al., 2019; Bai et al., 2020). Additionally, we performed GSVA analysis using “c7. immunesigdb.v2023.1. Hs.symbols set” to explore differences in immune-related processes between the clusters. In Cluster 1, colitis γδT cells from the colon, epithelial cells, and IL-10 STIM macrophage were activated. While in Cluster 2, incubation monocytes from tumor-bearing and IFN-γ Pam3Cys were more active (Figure 4C).

### Screening of gene modules

**FIGURE 5:**
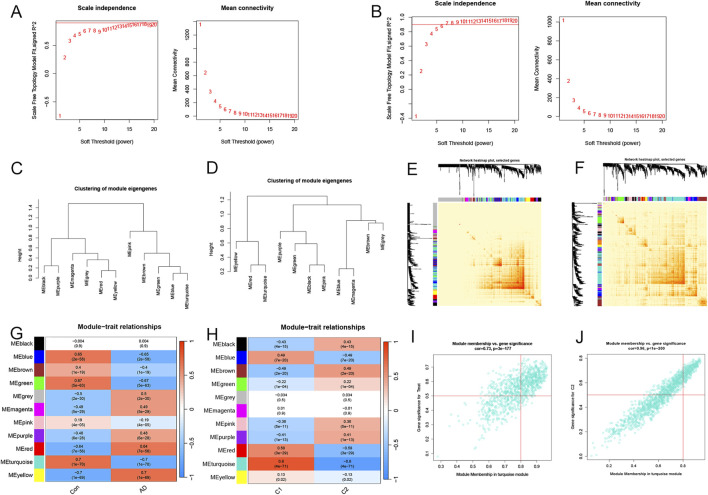
Screening significant gene modules by the WGCNA algorithm. Selecting the soft threshold powers in AD-traits **(A)** and cluster-traits **(B)**. Clusters of module eigengenes in the AD-related WGCNA **(C)** and cluster-related WGCNA **(D)**. **(E, F)** Representative heatmaps of correlations between the 11 blocks. **(G)** Correlation analysis of clinical condition and model eigengenes. Each column stands for a clinical condition, and each row for a module. **(H)** Relationships between the molecular clusters and model eigengenes. Each column stands for a cluster, and each row for a module. **(I)** A scatter plot between the significance of AD genes and module membership in the turquoise module. **(J)** A scatter plot between the significance of cluster-related genes and module membership in the turquoise module.

The WGCNA algorithm was utilized to build co-expression patterns and networks for identifying critical gene modules associated with AD and cuproptosis clusters. We calculated the gene expression variations in GSE33000 and chose the top 25% of genes with the largest variances for additional analysis. We determined *R* ^2^ = 0.9 and β = 16, 7 as the most appropriate soft threshold arguments for constructing the scale-free model networks (Figures 5A, B). Interestingly, a total of 22 modules (11 on both occasions) with different colors were defined as important, and the topological overlap matrix (TOM) of relevant genes was depicted in the heatmaps (Figures 5C–F). These genes in the 22 modules were then selected to explore the relevance and importance of module co-expression with clinical traits (Figures 5G, H). The two turquoise modules presented the most significant association with AD risk and cuproptosis clusters. They contained 759 hub genes and 297 hub genes, respectively (Supplementary Tables S4, S5). Moreover, the turquoise modules were positively correlated with module-related genes (cor = 0.73 and 0.96) (Figures 5I, J). |Gene Significance (GS)| > 0.5 and |Module Membership (MM)| > 0.8 were the standards for selecting hub genes.

### Construction of gene signature

**FIGURE 6:**
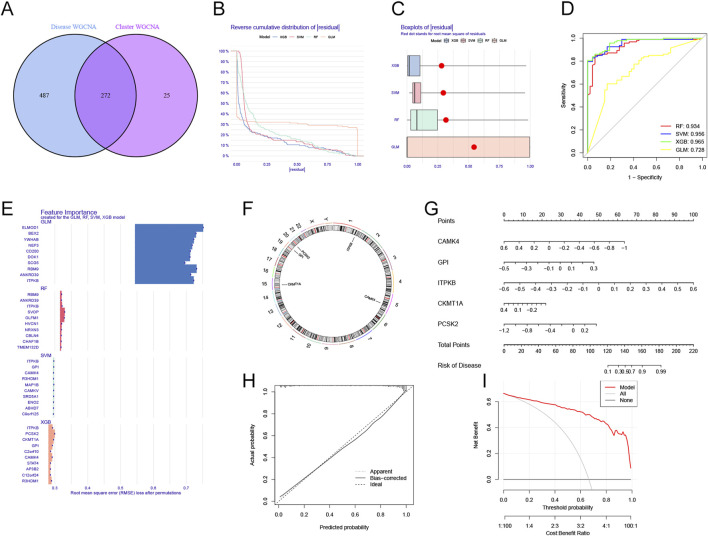
Construction of the prediction signature using machine learning techniques. **(A)** The intersections of disease-related WGCNA and cuproptosis cluster-related WGCNA. **(B)** Reverse cumulative residual distributions for the XGB, SVM, RF, and GLM models. **(C)** The residuals for each machine-learning model were shown in the boxplots. Red dots indicate the root mean square (RMSE). **(D)** The ROC analysis of machine learning models based on 5-fold cross-validations in the test queue. **(E)** The top 10 most important features in each machine learning model. **(F)** The locations of the five hub genes on 23 chromosomes. **(G)** Nomogram of the five hub genes. **(H)** Calibration curves revealing that the model may be an ideal predictive signature for AD. **(I)** DCA showing the predictive efficiency of the model.

In total, 272 genes were detected by analyzing the intersections of WGCNA analyses (Figure 6A; Supplementary Table S6). To further determine genes with the highest values for diagnosis, we developed four machine-learning models: Generalized Linear model (GLM), eXtreme Gradient Boosting (XGB), Random Forest model (RF), and Support Vector Machine model (SVM) based on the expression profiles of 272 hub genes. We randomized the data into a test cohort (30%) and a training cohort (70%). The results showed that XGB and SVM machine models exhibited relatively lower residuals (Figures 6B, C). We then assessed the four algorithms’ performance in the test cohort by computing receiver operating characteristic (ROC) profiles. The XGB machine model showed the most significant area under the ROC curves (AUC = 0.965) (Figure 6D). Finally, each model’s top 10 important genes were ranked according to root mean square error (RMSE) (Figure 6E). The top 5 most significant genes (*CAMK4, GPI, ITPKB, CKMT1A,* and *PCSK2*) in the XGB model were selected as predicted variables for AD. The locations of the genes on the human chromosomes are displayed in Figure 6F. We then constructed a nomogram to calculate the specific scores for each gene (Figure 6G). The calibration curves and decision curve analysis (DCA) were utilized to assess the performance of the nomogram model. The margin of error between the ideal risk and the actual risk for the AD cluster is small according to the calibration curve (Figure 6H). The DCA indicated that our model had high accuracy and could be used to inform clinical decisions (Figure 6I). We then evaluated the expression levels of five genes in the GSE33000 dataset. The results were consistent with the risk tendencies in the nomogram model (Supplementary Figure S1).

### Clinical correlations and external datasets

**FIGURE 7:**
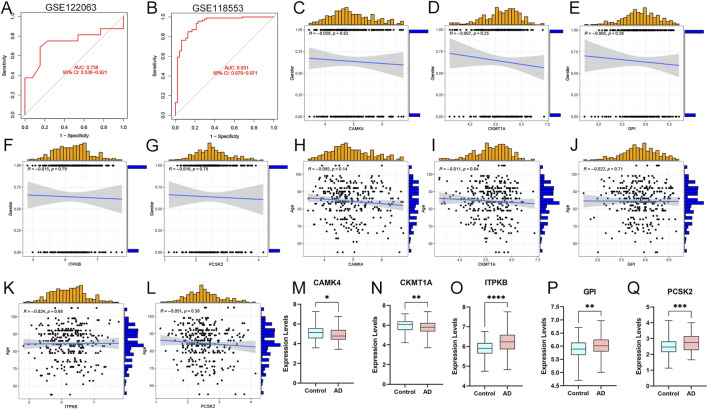
External validations and clinical correlation analysis of the model. **(A, B)** ROC analysis values in GSE122063 and GSE118553 datasets. **(C–G)** Correlation analysis between CAMK4, CKMT1A, GPI, ITPKB, PCSK2, and gender in AD patients. **(H–L)** Correlations between CAMK4, CKMT1A, GPI, ITPKB, PCSK2 and age. **(M–Q)** Relative expression levels of CAMK4, CKMT1A, GPI, ITPKB, and PCSK2 in AD and controls. *p < 0.05, **p < 0.01, ***p < 0.001.

We validated the prediction model using ROC curves on two external brain cortex datasets. The results revealed that our prediction model performed satisfactorily, with AUC values of 0.738 for the GSE122063 dataset and 0.931 for the GSE118553 dataset (Figures 7A, B). This suggests that our model is equally effective in distinguishing between AD patients and normal individuals. Moreover, we enrolled the five hub genes and an external dataset (GSE118553) to investigate the associations between our model and clinical traits, such as age and gender. The results revealed no significant relationships between the clinical characteristics and gene expression levels (Figures 7C–L). Finally, we used the external dataset GSE118553 to verify the expression levels of our predictive genes. The findings suggested that *CAMK4* and *CKMT1A* were significantly upregulated in the control groups, while *ITPKB*, *GPI*, and *PCSK2* were remarkably upregulated in the AD groups, consistent with our previous findings (Figures 7M–Q).

### Analysis of ceRNA networks and immune correlations

**FIGURE 8:**
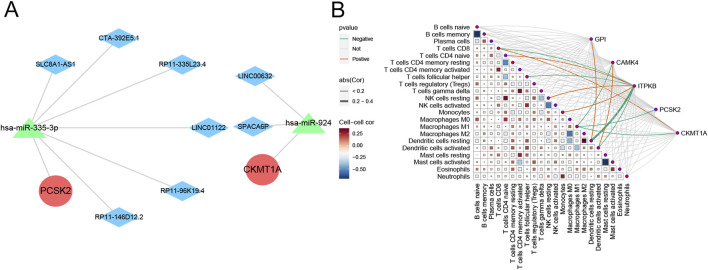
Prediction of ceRNA networks and immune correlations of the hub genes. **(A)** Prediction of ceRNA networks associated with hub genes. **(B)** Matrix diagram displaying the correlations between immune cells and hub genes.

We used the miRanda, miRDB, miRTarBase, and TargetScan databases to jointly screen potential miRNAs interacting with hub genes, then predicted the corresponding lncRNAs using the SpongeScan database, and finally constructed a ceRNA network using the Cytoscape software (version 3.8.2, Figure 8A). As shown in Figure 8B, PCSK2 was significantly negatively correlated with M1 macrophages. CKMT1A was positively associated with CD8 T cells and negatively correlated with M1 macrophages. ITPKB was strongly negatively correlated with resting dendritic cells, activated dendritic cells, follicular helper T cells, and CD8 T cells, but positively correlated with CD4 naïve T cells, resting NK cells, and monocytes. CAMK4 demonstrated close associations with M1 macrophages and activated dendritic cells. GPI showed significant positive relationship with resting dendritic cells.

### Networks of candidate drugs, interactive genes, and transcription factors

**FIGURE 9:**
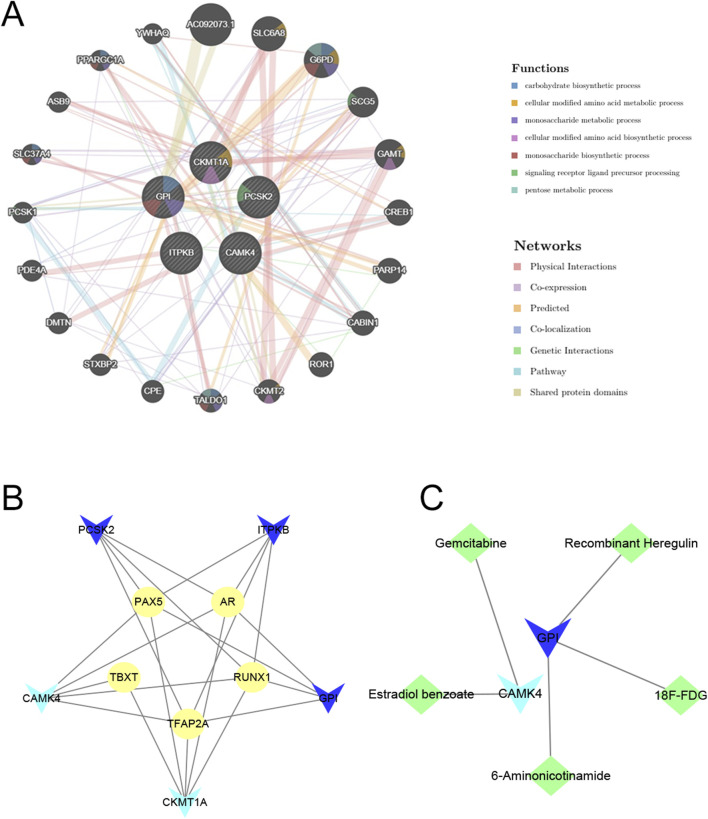
Prediction of interacting genes, transcription factors, and gene-targeted drugs. **(A)** Predicting interacting genes associated with hub genes. **(B)** The TF network based on marker genes. Yellow ellipse nodes denote transcription factors. **(C)** Interactions of genes and drugs. Green diamond nodes represent targeted drugs. Light blue V-shaped nodes for downregulated mRNAs, Dark blue V-shaped nodes for upregulated mRNAs.

The predicted three-dimensional structures and sequences of these five genes are shown in Supplementary Figure S2. To investigate potential interactions with the five hub genes, we used GeneMANIA to identify 20 candidate genes that may interact with them (Figure 9A). Interactions of drugs and TF regulators with hub genes are shown in Figures 9B, C. We identified 5 TF regulatory features. The five TFs were: AR (androgen receptor), PAX5 (paired box 5), RUNX1 (RUNX family transcription factor 1), TBXT (T-box transcription factor T), and TFAP2A (transcription factor AP-2 alpha). Surprisingly, all of these TFs could regulate the transcription process of each of the five hub genes, suggesting close correlations between the signatures of this model. Candidate drugs associated with hub genes were selected based on interaction scores and available literature (Table 3) (Zhou et al., 2014; He et al., 2014; Beatty et al., 2018).

**TABLE 3:** The candidate drugs interacted with hub genes.

Drug	Gene	Regulatory approval	Indication	Interaction score
6-Aminonicotinamide	GPI	Not Approved	—	7.864
Fluorodeoxyglucose F18	GPI	Approved	—	6.553
Recombinant Heregulin	GPI	Not Approved	—	5.617
Gemcitabine	CAMK4	Approved	Antineoplastic agent	0.324
Estradiol 3-Benzoate	CAMK4	Not Approved	—	5.898

### Validation of expression levels in AD mouse models

**FIGURE 10:**
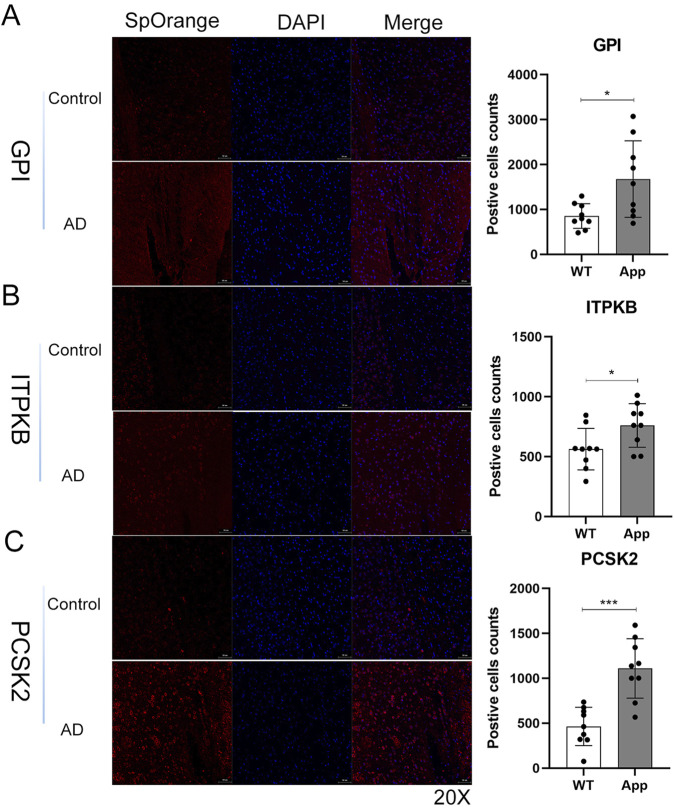
Validation of hub genes using immunofluorescence staining in mice brain tissues. **(A–C)** The representative images and qualifications of IF staining for GPI, ITPKB, and PCSK2 were shown (*, *p* < 0.05; ***, *p* < 0.001). GPI/ITPKB/PCSK2 (red), Nucleus (blue); Scale bar, 50um, (n = 3 per group).

We confirmed the five genes using immunofluorescence (IF) staining to validate our results. The expressions of *GPI, ITPKB,* and *PCSK2* were significantly increased in the cerebral cortex of AD groups compared to the control group (Figures 10A–C), which were consistent with our predictions. In contrast to our analyses, the expressions of *CKMT1A* and *CAMK4* were also upregulated in the AD group and the differences were not significant (Supplementary Figure S3). All IF staining results are displayed in Supplementary Figures S4–S8.

## Discussion

**FIGURE 11:**
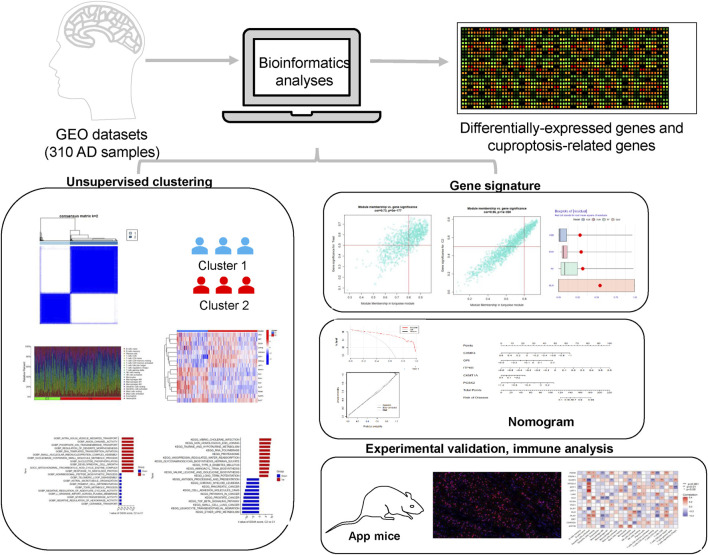
Flowchart of this study.

Growing evidence suggests that cuproptosis, a novel nonapoptotic, copper-dependent programmed cell death, plays a crucial role in neurodegenerative diseases (Mangalmurti and Lukens, 2022; Amtage et al., 2014). Nevertheless, its regulatory role has not been established, particularly in AD. In recent years, increasing advances have been applied in the treatment of AD, and the traditional histology-based classification has led to frequent drug resistance (Schneider et al., 2007; Dubois et al., 2021). In this research, we sought to explore the specific roles of CRGs in AD phenotypes and their relationship with the AD immune microenvironment, identify the hub genes, and probe the corresponding regulatory TFs and targeted drugs (Figure 11).

Our findings are expected to be applied in the clinical diagnosis and treatment of AD. The identification of CRGs and the subsequent classification of AD patients into two distinct molecular clusters provide valuable information for understanding the heterogeneity of AD. Functional analyses showed DECRGs were significantly enriched in the mitochondrial redox regulation metabolisms. This discovery aligns partially with the research of Tsvetkov et al. (Tsvetkov et al., 2022).

The etiology of AD is complex and involves many factors, and recent findings have suggested a critical role for immunity in its pathogenesis (Si et al., 2023; Wightman et al., 2021). AD samples contained elevated infiltration levels of resting NK cells, naïve CD4^+^ T cells, resting CD4^+^ memory T cells, M2 macrophages, neutrophils, and monocytes, consistent with previous studies (Saresella et al., 2011; Zhao et al., 2022; Khan et al., 2021; Borkowski et al., 2021). These findings suggest that dysregulation of infiltration in the immune microenvironment plays a critical role in AD.

Moreover, previous studies revealed the involvement of cuproptosis in the pathogenesis of AD neuroinflammation (Mangalmurti and Lukens, 2022). Therefore, we systematically evaluated the correlations between the 16 DECRGs and 22 immune infiltrating cells in AD. *PDHB* and *DLST* were significantly associated with most of the immune infiltrating cells.

Compared with Cluster 2, the abundance of *DLST*, *PDHB*, *NLRP3*, *DLAT*, *PDHA1*, *MTF1*, *FDX1*, *DLD*, and *GLS* was significantly elevated in Cluster 1. Cluster 1 presented a higher level of immune infiltration and was relatively dominant in neuroinflammation. GSVA results suggested that Cluster 1 was mainly involved in the immune-associated pathways, such as antigen processing and presentation, TGF-β signaling pathway, and leukocyte trans-endothelial migration. In contrast, Cluster 2 was primarily characterized by RNA polymerase and long-term potentiation. TGF-β signaling pathway has been found to be essential for the activation and differentiation of T and B cells, while neuroinflammation has been implicated as a critical factor in the pathogenesis of AD (possibly in its early stages) (Du et al., 2021; Lee, 2020; Ni and Lynch, 2020). Taken together, we hypothesize that Cluster 1 may harbor more activated B cells and T cells that contribute to the development of AD and, therefore, have a worse outcome than Cluster 2. These findings suggest that immune modulation may play a critical role in the progression of AD, offering new avenues for targeted therapeutic interventions.

Recently, an increasing number of studies have used machine-learning models to predict morbidity (Alatrany et al., 2021; Singhania et al., 2021), and these studies suggested that multivariate analyses assessed the relationships between variables and provided more reliable results with lower error rates than univariate analyses. We investigated the predictive properties of machine learning models (SVM, RF, XGB, and GLM) and constructed an XGB prediction model with the highest fidelity in the test cohort (AUC = 0.965). We then selected the five most significant variables (*CAMK4, GPI, ITPKB, CKMT1A,* and *PCSK2*) to establish a 5-gene nomogram. Immunofluorescence staining was conducted to validate our findings, which were consistent with previous analyses. These five genes were assigned different points. The points were added together to give the total points. The AD risk is less than 0.1 if the total points do not exceed 120, and greater than 0.99 if the total points exceed 180.

Hyperphosphorylated tau proteins are the primary component of neurogenic fiber tangles in the brains of AD patients. Yet, the mechanism is incompletely understood. Wei et al. concluded that intracellular accumulation of phosphorylated tau triggered nuclear Ca2^+^/CAMK4 signaling, exacerbating tau hyperphosphorylation (Wei et al., 2018). In contrast, Yin et al. found that Ca2^+^/CAMK4 was inhibited when human wild-type full-length tau (hTau) accumulated intracellularly (Yin et al., 2016). Calcium -dependent protein kinase IV (*CAMK4*) is a multifunctional enzyme engaged in regulating multiple cellular processes, including memory formation, neuronal health, and calcium signaling (Bito and Takemoto-Kimura, 2003). Specifically, *CAMK4* is essential for regulating synaptic plasticity, especially long-term potentiation (LTP), which is critical for memory and learning. Studies have shown that *CAMK4* may protect neurons by regulating the expression of genes that support neuronal survival. In AD, the neuroprotective effects of *CAMK4* might be impaired, resulting in neurons being more susceptible to mitochondrial dysfunction, oxidative stress, and neuroinflammation (Bell et al., 2013). Similarly, *ITPKB* is an enzyme involved in the regulation of intracellular calcium (Ca^2^⁺) signaling, which is severely disrupted in AD (Schienle and Scharmüller, 2013). By regulating calcium release through modulation of IP3, *ITPKB* influences memory processes, cell survival, and synaptic plasticity (Sun et al., 2017). Dysregulation of *ITPKB* may exacerbate calcium imbalances induced by amyloid-β pathology, leading to neurodegeneration, neuroinflammation, and synaptic dysfunction in AD (Kalinec et al., 2017). Previous studies have also found that *ITPKB* was an essential regulator of neuronal apoptosis and tau phosphorylation in Alzheimer’s disease, suggesting that *ITPKB* may be a novel target for mitigating pathological changes in AD (Stygelbout et al., 2014; Salta et al., 2016).

Glycolysis is found to be the most significant overexpression of gene onto-biological processes associated with altered protein aggregation between AD and control patients. Glucose-6 phosphate isomerase (*GPI*) is the predominant insoluble protein identified by proteomics and increased in all insoluble fractions of AD brain samples as verified by Western blotting. (Kepchia et al., 2020). Moreover, GPI-anchored proteins perform important roles in synaptic plasticity, neuronal signaling, and protection against AD-associated toxicity. Disruption of GPI biosynthesis or function might contribute to the psychophysiology of AD, rendering this pathway an emerging area of interest for potential therapeutic interventions (Liu et al., 2022). Understanding how GPI proteins interact with AD pathology could provide new insights into novel therapeutic strategies and mechanisms of disease progression. *CKMT1A* is a mitochondrial creatine kinase involved in multiple gene expression regulation and signaling pathways (Gobinath et al., 2017). Its dysfunction is closely linked to mitochondrial abnormalities observed in AD. *CKMT1A* helps neurons meet their high energy demands by regulating the creatine-phosphocreatine shuttle (Frederiksen et al., 2018). However, in AD, oxidative stress, Aβ toxicity, and mitochondrial dysfunction impair the ability of *CKMT1A* to maintain energy homeostasis, leading to cognitive decline, neurodegeneration, and synaptic failure (Cha et al., 2019). Treatment strategies that support *CKMT1A* function may offer potential approaches to slow AD progression.

*PCSK2* is a member of the proprotein convertase family involved in key processes associated with AD, particularly neuroinflammatory regulation and processing of amyloid precursor proteins (Mukherjee et al., 2017). By affecting synaptic function and Aβ production, *PCSK2* may play an important role in the pathogenesis of AD. Interestingly, in AD patients, levels of *PCSK2* were inversely correlated with levels of neurodegeneration markers (Barranco et al., 2021). Further studies are required to elucidate its exact mechanism and explore its potential as a therapeutic target for AD.

The accuracy of this model was well validated in two external datasets (AUC = 0.931 and 0.738), suggesting that it could be used as a non-invasive biomarker for early detection of AD. This genetic signature could be used to complement existing diagnostic tools, reducing the need for invasive procedures such as lumbar puncture or advanced imaging techniques. Clinicians could use this genetic signature in a clinical setting to better stratify patients, monitor disease progression and develop personalized treatment strategies based on AD molecular subtypes. Moreover, the expression levels of five genes were again verified in GSE33000 and GSE118553 datasets. We also investigated the associations between the five hub genes and clinical characteristics. The TF regulatory network and gene-drug interactions provided novel insights into AD pathogenesis and drug mining.

In addition, the immune-related pathways identified in our study, such as the TGF-β signaling pathway and immune cell regulation, are already being explored in other neurodegenerative diseases. Thus, our findings may support the repurposing of existing immunomodulatory therapies for AD patients, particularly those in Cluster 1, who may benefit from treatments targeting immune regulation. However, further clinical trials are needed to validate these potential therapeutic applications and determine their efficacy in different AD subgroups.

Still, there are some limitations to our study. First, the data sources were from online databases, and input mistakes couldn't be estimated. Secondly, though we included 19 generally recognized cuproptosis-related genes, there is still a need to include more newly identified CRGs. Although the mouse experiments confirmed the results to some extent, we are currently unable to obtain sufficient clinical AD samples for large-scale validation. Finally, further basic experiments are necessary to explore the exact mechanisms underlying the involvement of these genes in cuproptosis related to AD.

## Conclusion

In summary, this study revealed close associations between infiltrating immune cells and CRGs, emphasizing the heterogeneous nature of AD molecular clusters and immune responses. This model highlights the potential therapeutic implications of targeting cuproptosis and immune pathways in AD, providing a foundation for personalized treatment strategies.

## Data Availability

The original contributions presented in the study are included in the article/Supplementary Material, further inquiries can be directed to the corresponding authors.
