# Supplementary material for: Novel cuproptosis metabolism-related molecular clusters and diagnostic signature for Alzheimer’s disease
Source: Front Mol Biosci. 2024 Oct 24;11:1478611. doi: 10.3389/fmolb.2024.1478611 (PMC11540791; doi:10.3389/fmolb.2024.1478611)
Supplement: Supplementary file 3 [file Table1.docx]

Supplementary Table 1. The specific information of three cortex datasets.

| **Dataset** | **Platform** | **AD samples** | **Normal samples** | **Resource** |
| --- | --- | --- | --- | --- |
| GSE122063 | GPL16699(Agilent-039494 SurePrint G3 Human GE v2 8x60K Microarray) | 92 | 44 | Cerebral cortex |
| GSE33000 | GPL4372(Rosetta/Merck Human 44k 1.1 microarray) | 310 | 157 | Cerebral cortex |
| GSE118553 | GPL10558(Illumina HumanHT-12 V4.0 expression beadchip) | 301 | 100 | Cerebral cortex |

AD, Alzheimer's disease
