# Supplementary material for: Novel cuproptosis metabolism-related molecular clusters and diagnostic signature for Alzheimer’s disease
Source: Front Mol Biosci. 2024 Oct 24;11:1478611. doi: 10.3389/fmolb.2024.1478611 (PMC11540791; doi:10.3389/fmolb.2024.1478611)

Figure S1. The expression levels of five risk genes in the GSE33000 presented with the boxplot. Of these, GPI, ITPKB, and PCSK2 were significantly upregulated in AD groups, while CAMK4 and CKMT1A were downregulated. **, p< 0.01; ****, p< 0.001.

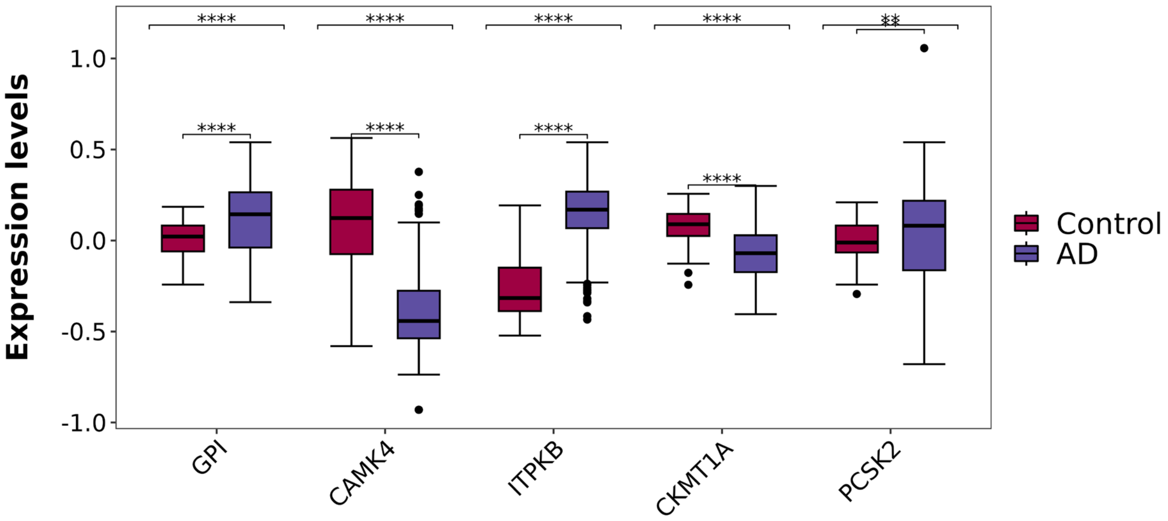

Figure S2. The predicted three-dimensional structures and sequences of the risk genes.

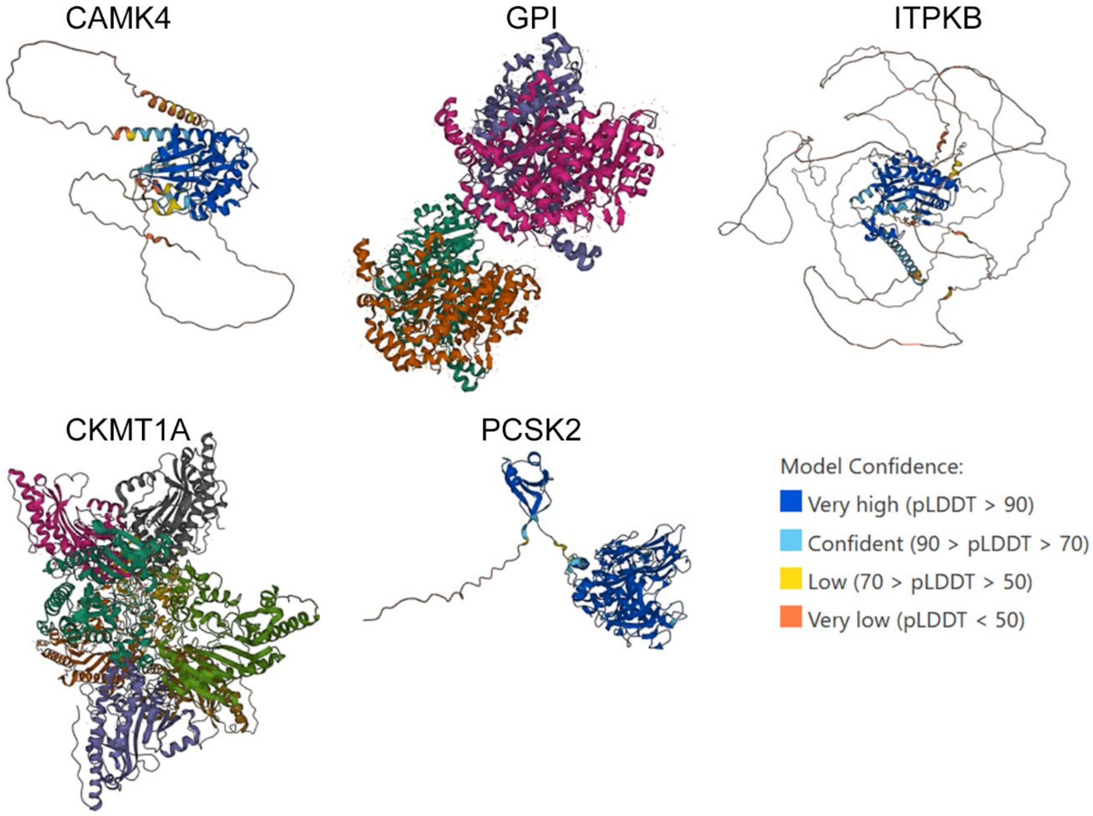

Figure S3. The representative images and qualifications of IF staining for CAMK4 and CKMT1A.

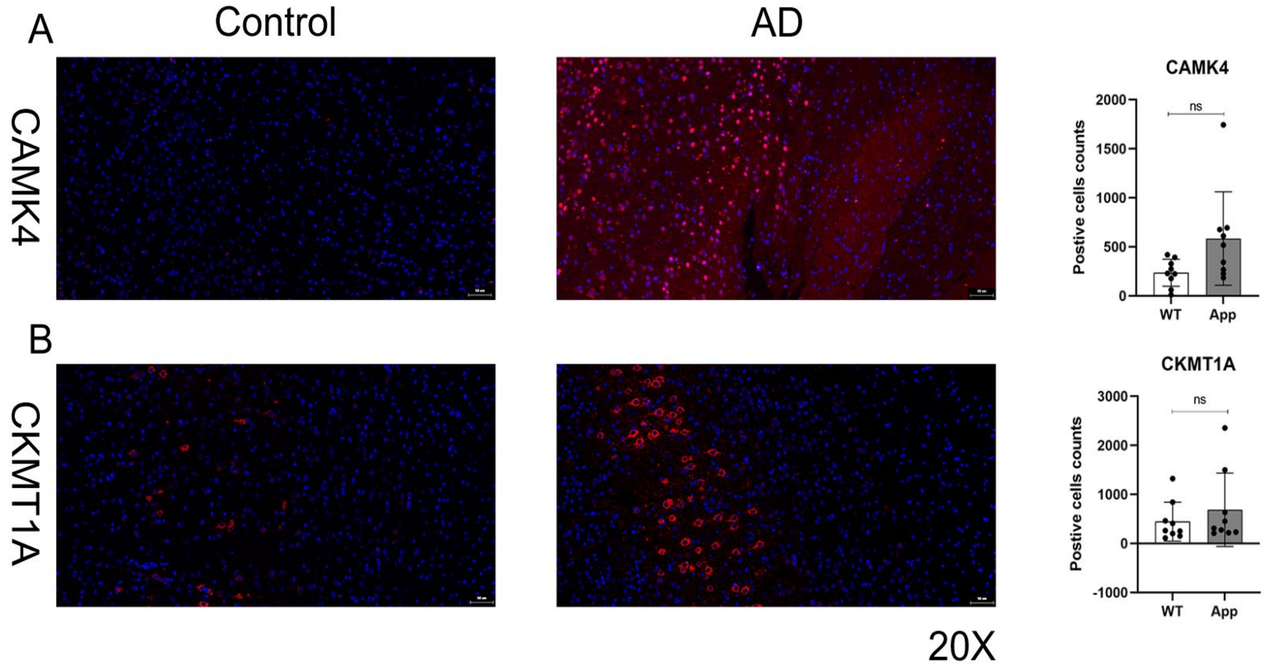

Figure S4. The immunofluorescence imaging of the whole brain for CAMK4.

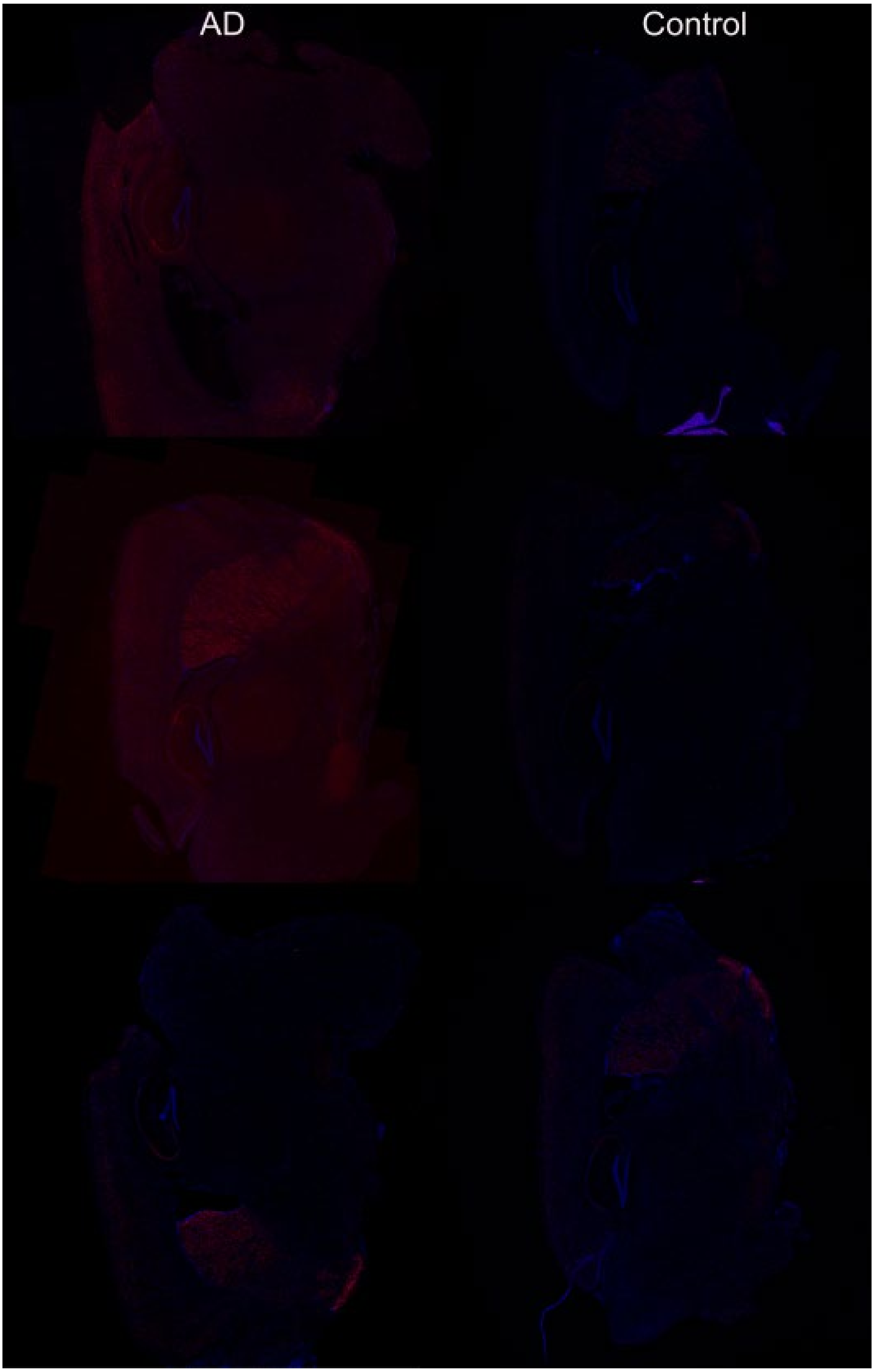

Figure S5. The immunofluorescence imaging of the whole brain for CKMT1A.

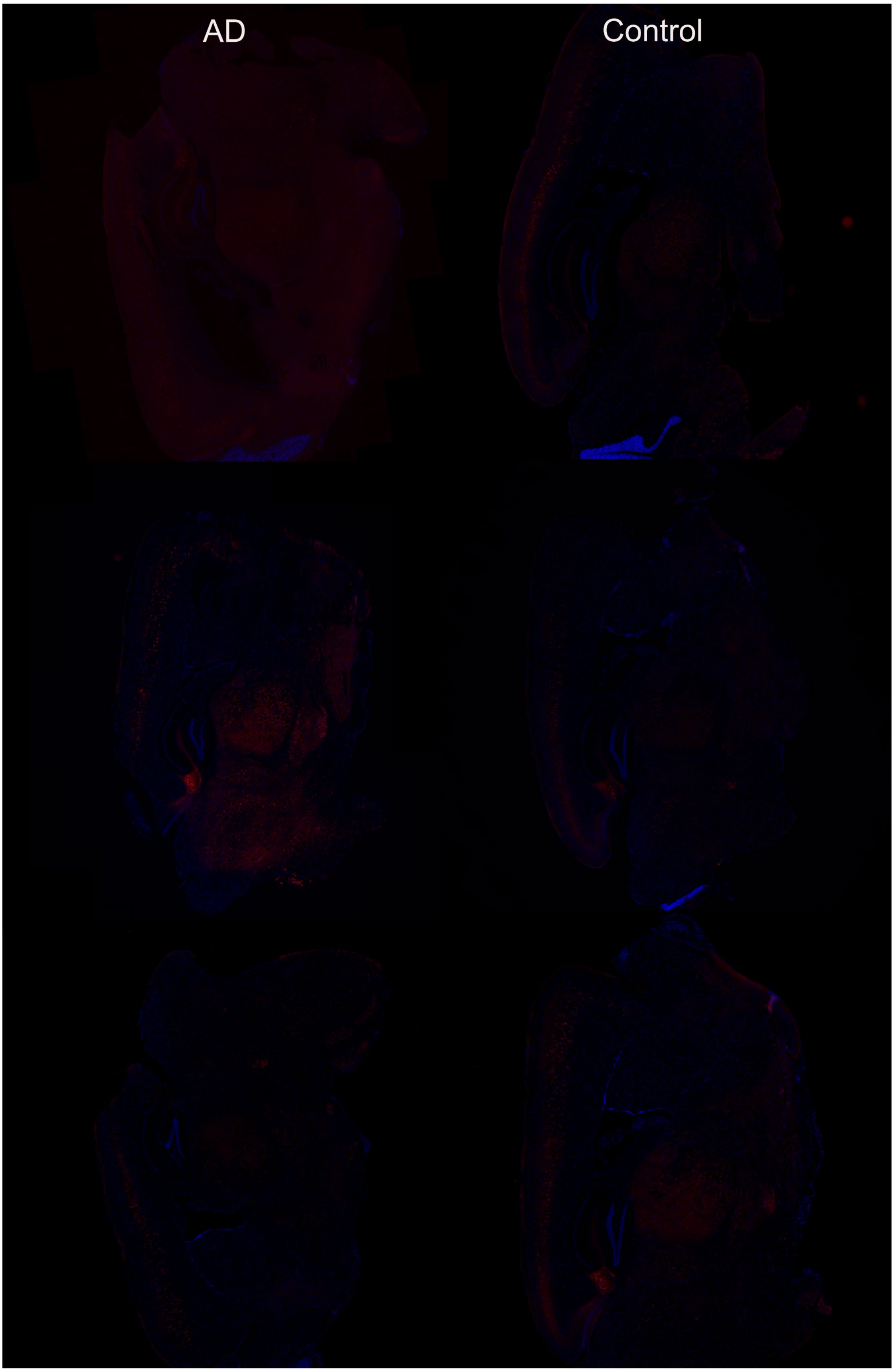

Figure S6. The immunofluorescence imaging of the whole brain for GPI.

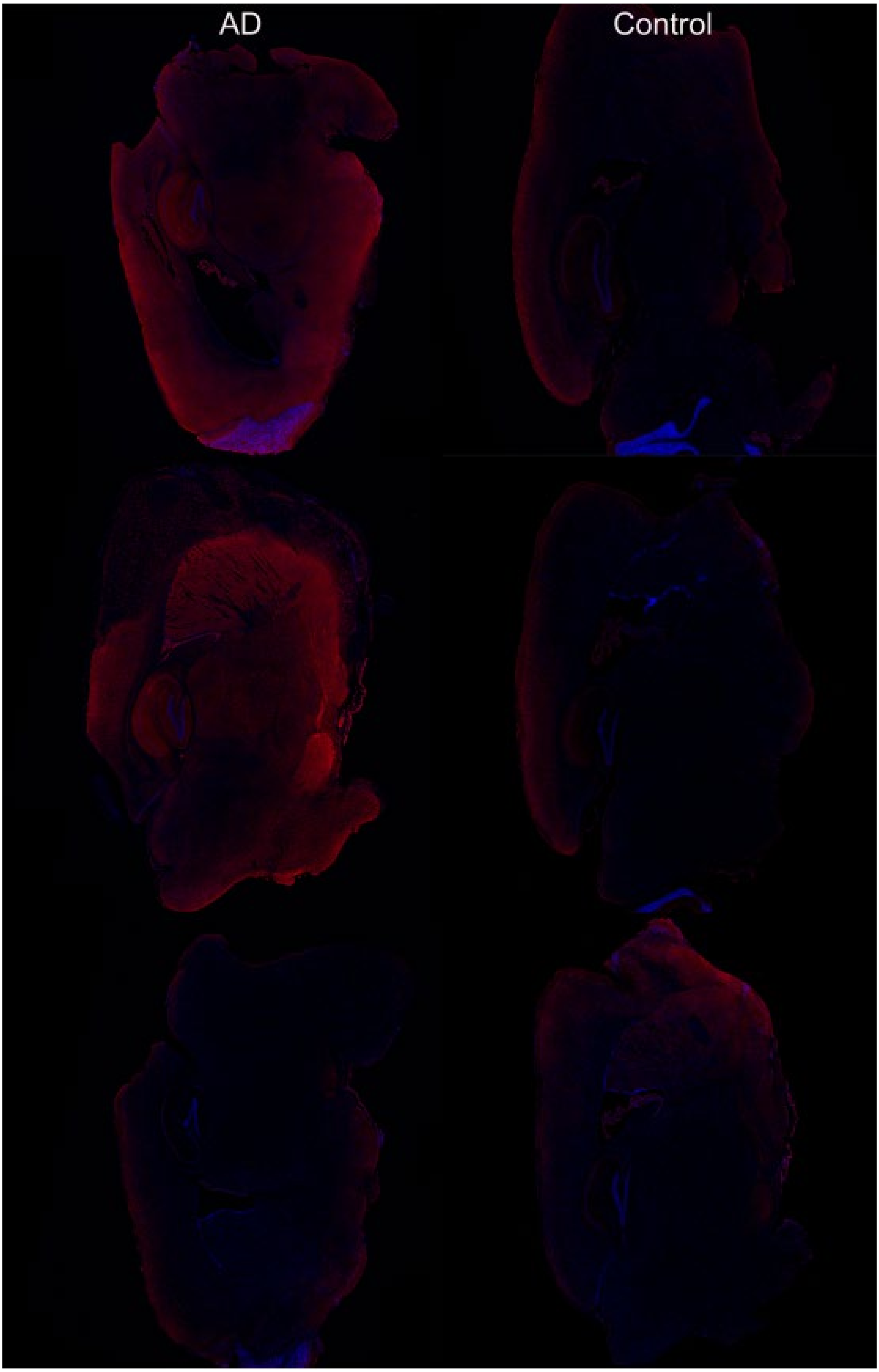

Figure S7. The immunofluorescence imaging of the whole brain for ITPKB.

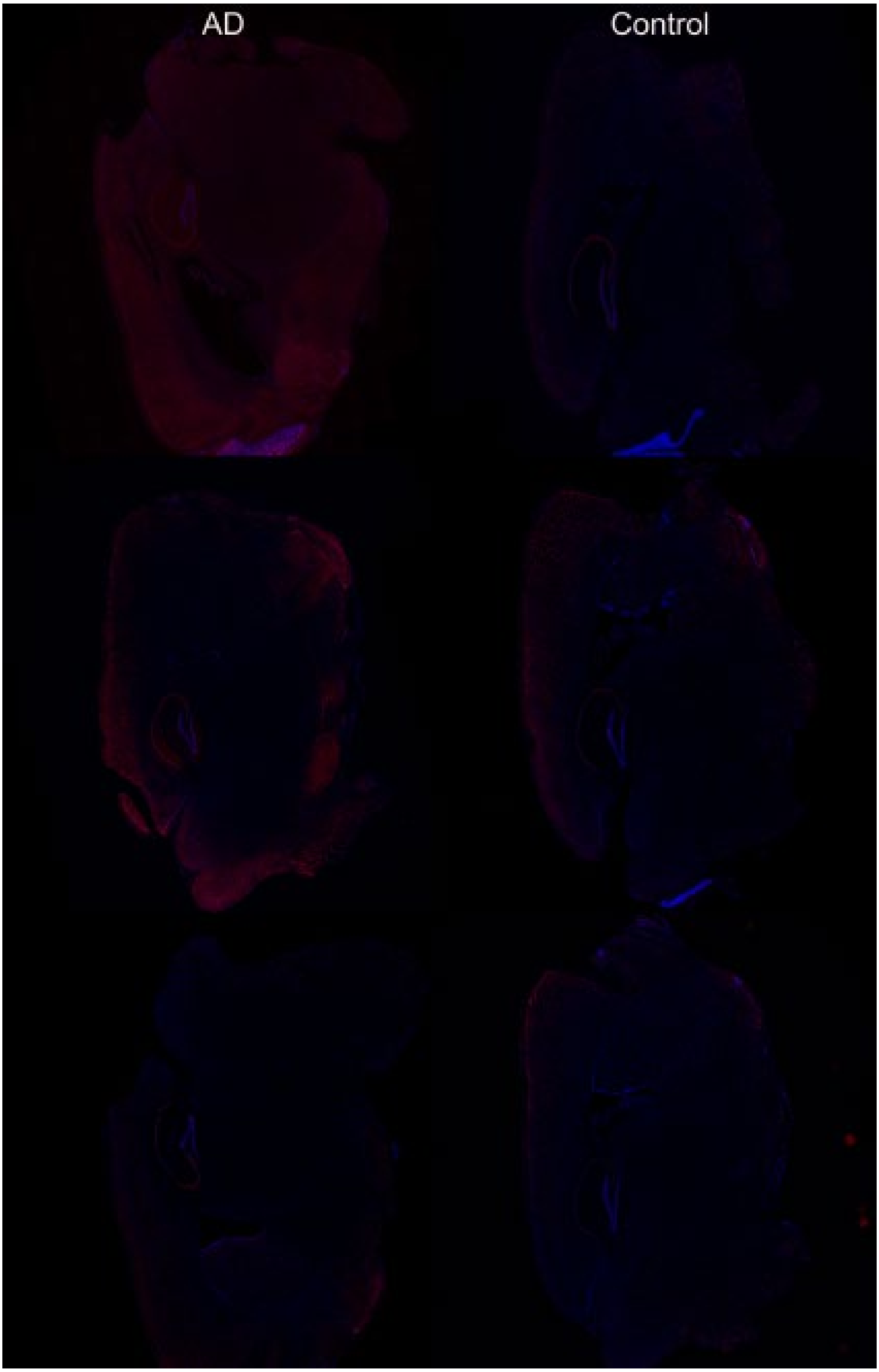

Figure S8. The immunofluorescence imaging of the whole brain for PCSK2.

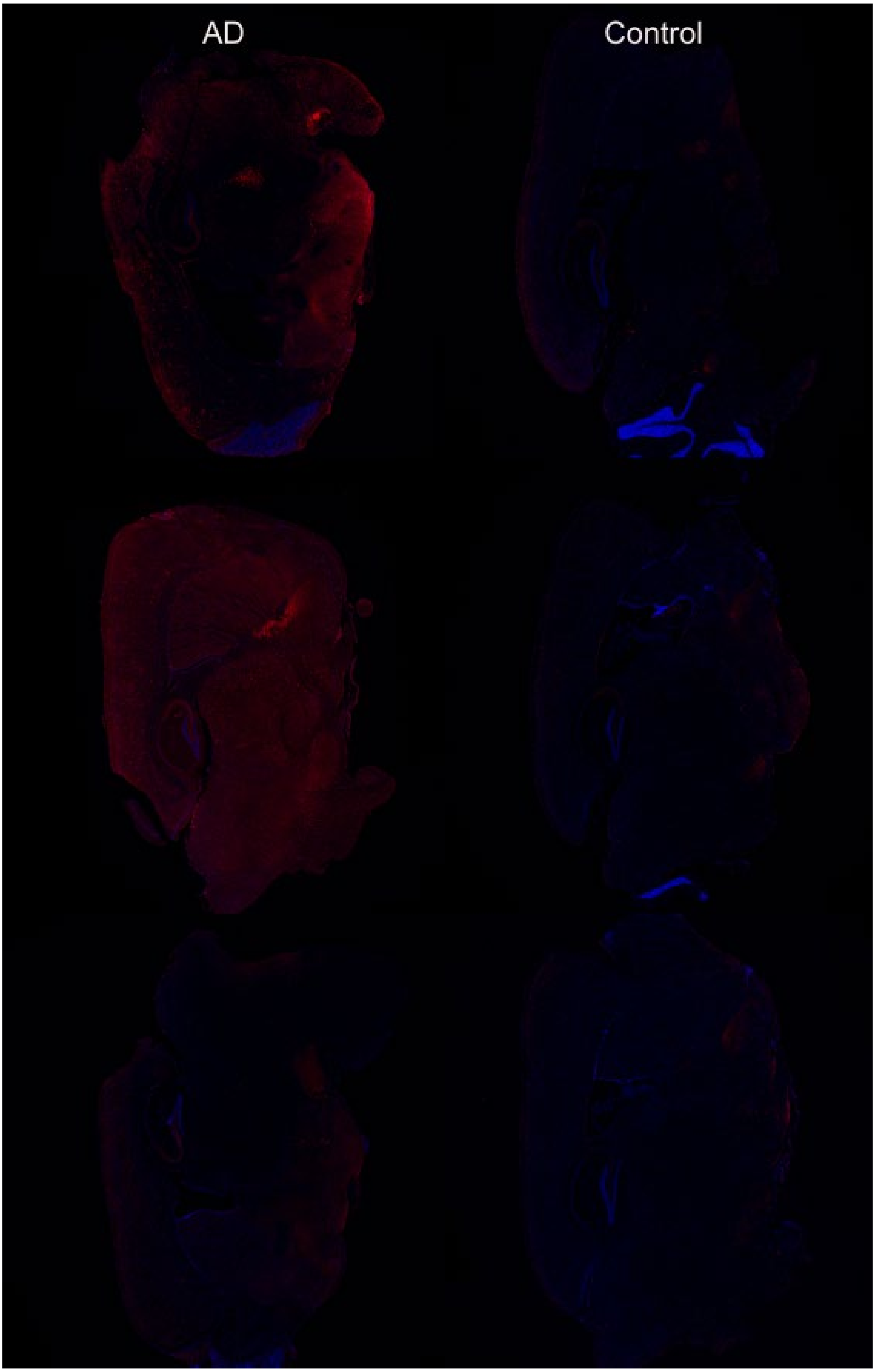

Supplement: Supplementary file 7 [file Image1.pdf]
